# Personal Protection Equipment Training as a Virtual Reality Game in Immersive Environments: Development Study and Pilot Randomized Controlled Trial

**DOI:** 10.2196/69021

**Published:** 2025-03-20

**Authors:** Liang Zhou, Haoyang Liu, Mengjie Fan, Jiahao Liu, Xingyao Yu, Xintian Zhao, Shaoxing Zhang

**Affiliations:** 1 Institute of Medical Technology, Peking University Health Science Center, Beijing, China; 2 National Institute of Health Data Science, Peking University, Beijing, China; 3 Beijing Forestry University, Beijing, China; 4 Visualization Research Center (VISUS), University of Stuttgart, Stuttgart, Germany; 5 Department of Healthcare-Associated Infection Management, Peking University Third Hospital, Beijing, China; 6 Department of Otolaryngological, Peking University Third Hospital, 49 Huayuanbei Road, Beijing, 100191, China, 86 13811403347

**Keywords:** virtual reality training, nosocomial infections control, visualization, human computer interaction, personal protection equipment, PPE

## Abstract

**Background:**

Proper donning and doffing of personal protection equipment (PPE) and hand hygiene in the correct spatial context of a health facility is important for the prevention and control of nosocomial infections. On-site training is difficult due to the potential infectious risks and shortages of PPE, whereas video-based training lacks immersion which is vital for the familiarization of the environment. Virtual reality (VR) training can support the repeated practice of PPE donning and doffing in an immersive environment that simulates a realistic configuration of a health facility.

**Objective:**

This study aims to develop and evaluate a VR simulation focusing on the correct event order of PPE donning and doffing, that is, the item and hand hygiene order in the donning and doffing process but not the detailed steps of how to don and doff an item, in an immersive environment that replicates the spatial zoning of a hospital. The VR method should be generic and support customizable sequencing of PPE donning and doffing.

**Methods:**

An immersive VR PPE training tool was developed by computer scientists and medical experts. The effectiveness of the immersive VR method versus video-based learning was tested in a pilot study as a randomized controlled trial (N=32: VR group, n=16; video-based training, n=16) using questionnaires on spatial-aware event order memorization questions, usability, and task workload. Trajectories of participants in the immersive environment were also recorded for behavior analysis and potential improvements of the real environment of the health facility.

**Results:**

Comparable sequence memorization scores (VR mean 79.38, SD 12.90 vs video mean 74.38, SD 17.88; *P*=.37) as well as National Aeronautics and Space Administration Task Load Index scores (VR mean 42.9, SD 13.01 vs video mean 51.50, SD 20.44; *P*=.16) were observed. The VR group had an above-average usability in the System Usability Scale (mean 74.78>70.0) and was significantly better than the video group (VR mean 74.78, SD 13.58 vs video mean 57.73, SD 21.13; *P*=.009). The analysis and visualization of trajectories revealed a positive correlation between the length of trajectories and the completion time, but neither correlated to the accuracy of the memorization task. Further user feedback indicated a preference for the VR method over the video-based method. Limitations of and suggestions for improvements in the study were also identified.

**Conclusions:**

A new immersive VR PPE training method was developed and evaluated against the video-based training. Results of the pilot study indicate that the VR method provides training quality comparable to video-based training and is more usable. In addition, the immersive experience of realistic settings and the flexibility of training configurations make the VR method a promising alternative to video instructions.

## Introduction

### Overview

Nosocomial infections are infections acquired within health care facilities. Such infections not only extend hospital stays and treatments of a patient but can also result in severe complications and even death [1,2]. Moreover, health care workers face an increased risk of infection due to cross-contamination that can escalate workload and disrupt health care services. Implementing preventive measures such as proper hand hygiene, disinfection protocols, appropriate personal protection equipment (PPE) use, and clean environment maintenance can significantly reduce the risk of such infections [3-5]. Notably, the availability, selection, and proper training of PPE is critical for reducing the risks of nosocomial infections [5,6]. The order of and the correct handling during donning and doffing of PPE is critical for reducing infections. It is known that a high risk of contamination exists during doffing [7].

On top of that, infection control protocols of a health care facility often involve spatial division for different protection and disinfection levels, for example, the operation room requires a higher PPE protection level than that of the outpatient building. In the case of a pandemic such as COVID-19, the “three areas and two corridors” division is used for health care facilities [8,9]. There, PPE donning and doffing have to be done in specific orders in specific regions to ensure the isolation of contaminated regions from clean regions.

### PPE Training in Health Facilities

PPE training is typically organized by each health facility with in-person demonstrations and lecturing. A major benefit of in-person training in a health facility is that personnel can be trained in a real spatial context. However, training in the real environment has severe shortcomings: First, the risk of the trainee being infected and the contamination of the uninfected regions; second, the lack of repeated practice due to the potential shortage of PPE. To reduce differences in training and infectious risks, standardized PPE training videos are available. Participants passively acquire the information as the trainer presents lecture slides or demonstrates the procedure, and need to practice on their own with real PPE equipment that is sometimes in shortage. Simulation-based education is an alternative to lectures as it is effective for PPE training with reduced cognitive load [10,11].

### Infection Control Training in Virtual Reality

Virtual reality (VR) and augmented reality are extensively used in medical training for a wide range of procedures, for example, surgery [12,13], needle insertion [14,15], and ultrasound [16]. Compared with augmented reality, VR provides a more immersive experience, saves training costs, and avoids exposure risks and resource consumption in real environments [17,18]. Therefore, we decide to devise our training method in VR.

VR methods are available for infection control training for various scenarios [19-24]. A VR infection control simulation program is available for training nurses to help children patients with COVID-19 [22]. There, donning and doffing of PPE, and the treatment of patients are simulated. However, the immersive environment does not focus on the spatial arrangements of zones of a real hospital; furthermore, the control group of this study did not receive video-based or conventional training but only did a questionnaire survey. For the management of patients with COVID-19, VR methods are developed for the infection control and diagnosis process [19] and proper case handling [20].

Recently, a VR simulator has been available for training detailed steps in the donning and doffing process of PPE [25]. The simulator is further extended by the use of reinforcement learning for training and interactive reinforcement learning for assessing the procedure of donning and doffing PPE [26].

Evidence is found for VR-based PPE donning and doffing training to be superior to traditional methods. Better performance is achieved with VR-based PPE donning and doffing training as higher performance scores than the traditional e-learning method are yielded [27]. Immersive 360-degree VR training is found to have comparable effectiveness to face-to-face training and is superior to video training [28].

VR is also used for infection control for operations in specific areas of a health care facility, such as preventing surgical-site infections in the operating room [23], and in neonatal intensive care units [21]. Hand hygiene and PPE donning are taught through 360-degree videos in VR for infection control training which is shown to be superior to traditional slideshow lecturing [24]. However, no work is currently available for interactive training of PPE donning and doffing embedded in an immersive environment with a spatial division for infection control that simulates a real health care facility.

### Our VR Method for PPE Training in a Spatial Context

In this paper, we propose an immersive PPE training method in VR with simulated spatial division of a hospital for nosocomial infection prevention to address this issue. Our work is a close collaboration with medical professionals who provided health care support for the Winter Olympic Games 2022 during the COVID-19 pandemic. Our immersive training method involves the modeling of the workspace, the avatar, and PPE donning and doffing logic based on the actual setting of the supporting hospital of the Winter Olympic Games. In a pilot study of 32 participants, we compared our new method to traditional video-based instruction learning. In addition to traditional knowledge tests and subjective evaluations, the study also recorded the trajectories of participants in the immersive environment for behavior analysis and potential improvements of the simulation and the real health facility. Results demonstrate that our VR method has a comparable training quality in terms of memorizing PPE donning and doffing orders with better subjective evaluations.

The main benefit of our method is that it allows the practice of PPE donning and doffing according to the infection prevention and control protocols immersively in a virtual environment that resembles the actual zoning of a hospital without wasting PPE which can be potentially scarce. Another benefit is that our method allows for easy and flexible editing of the donning and doffing sequence with a spatial context that adapts to the actual situation of a health facility.

## Methods

### Immersive VR PPE Training Method

#### Overview

An immersive PPE training method as a mini VR game was developed as a collaboration between computer science experts and medical experts. Based on the requirement analysis, we designed the generic donning and doffing sequence model, the virtual environments, the avatar, and the training logic. Our VR training method was implemented by computer scientists in our group as a Unity application.

#### Requirement Analysis

The requirements were initially proposed by medical experts who took the PPE training in a support hospital for the Winter Olympic Games 2022, and finalized through iterative discussions between computer science experts and medical experts. The hospital was under a high infection prevention and control level to ensure the safety and high-quality health care services of the Games. Medical experts identified 2 challenges: first, PPE includes many items and they have to be donned and doffed properly in the correct order; second, the doffing has to be done in the correct zones that need to be familiarized. Specifically, in this case, PPE includes medical masks, N-95 masks, protection suits, gowns, inner gloves, outer gloves, goggles, face shields, and shoe covers. The hospital was spatially divided using the “three zones and two corridors” protocol and several rooms were allocated for different steps of doffing.

After discussions, both the medical and computer science experts agreed that an interactive VR method should be designed as it can support the repeated practice of donning and doffing and an immersive experience in an environment that simulates the hospital. The requirements of the VR method are as follows:

- Requirement 1 (R1): focus on the practice of PPE donning and doffing sequence.
- Requirement 2 (R2): Simulation of the spatial division of the hospital relevant to PPE donning and doffing.
- Requirement 3 (R3): configurable donning and doffing order for flexibility and extensibility.

Therefore, we conceptualized the VR method as a mini-game that guides users to learn the correct ordering. The trainee starts from the donning zone, and she or he has to follow the correct donning and hand sanitation order to get access to the work zone. The door unlocks only after the prescribed donning sequence is successfully performed without any error. Next, in the same fashion, the trainee has to correctly follow the doffing order with thorough hand sanitation to proceed to subsequent doffing zones to finish the game.

#### Generic Donning and Doffing Sequence Modeling

**Figure 1.**
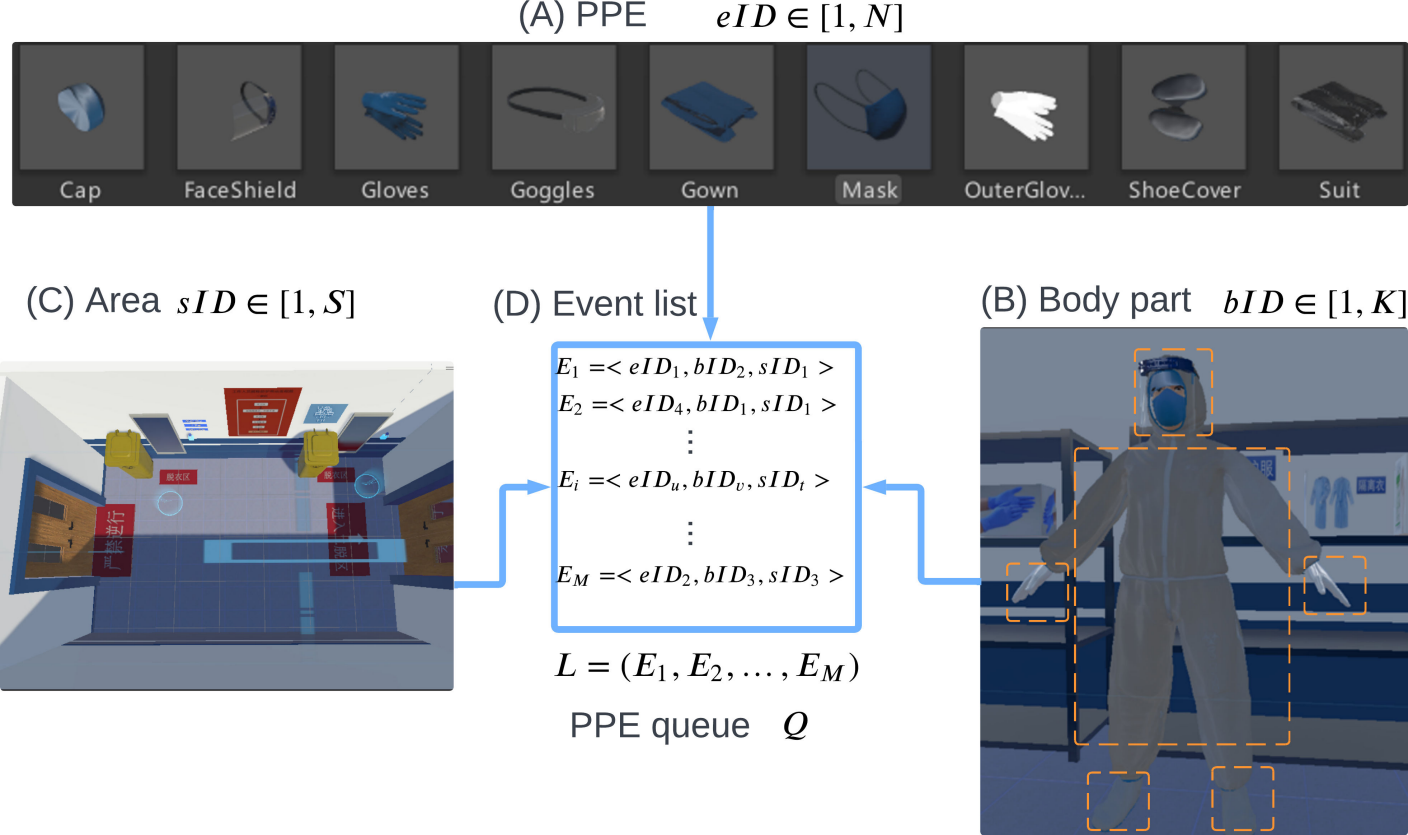
The generic donning and doffing sequence model. PPE: personal protection equipment.

A generic donning and doffing sequence model is devised to enable flexible customization of PPE simulation training (R3). As shown in Figure 1, each PPE is associated with an eID, body part identifier bID, and region identifier sID. The donning and doffing event triples $E_{i}$ are recorded in a list *L*:


L=(E_1,E_2,...,E_M),

$$E_{i}=<eID_{u},bID_{v},sID_{t}>,$$

where i∈[1,M],u∈[1,N],v∈[1,K],s∈[1,S].

Here, M,N,K, and S are the total steps of donning and doffing, the number of types of PPE, the number of body parts, and the number of zones, respectively. To ensure that all PPE is properly doffed, a queue Q is used to record the PPE status. If a piece of equipment is donned, it is enqueued into Q, and is dequeued if the equipment is doffed, and Q should be empty at the end of the training. The eID and bID in an event were prebounded so that the PPE can only be put on or removed when correctly corresponds to the body position and the collision detection has been passed. Therefore, the trainer only needs to set the sequence of events in corresponding areas to create a customized donning and doffing sequence according to the actual situation. The sequence in this paper follows the actual order in the supporting hospital during the 2022 Winter Olympics Games.

#### Virtual Environment Modeling

**Figure 2.**
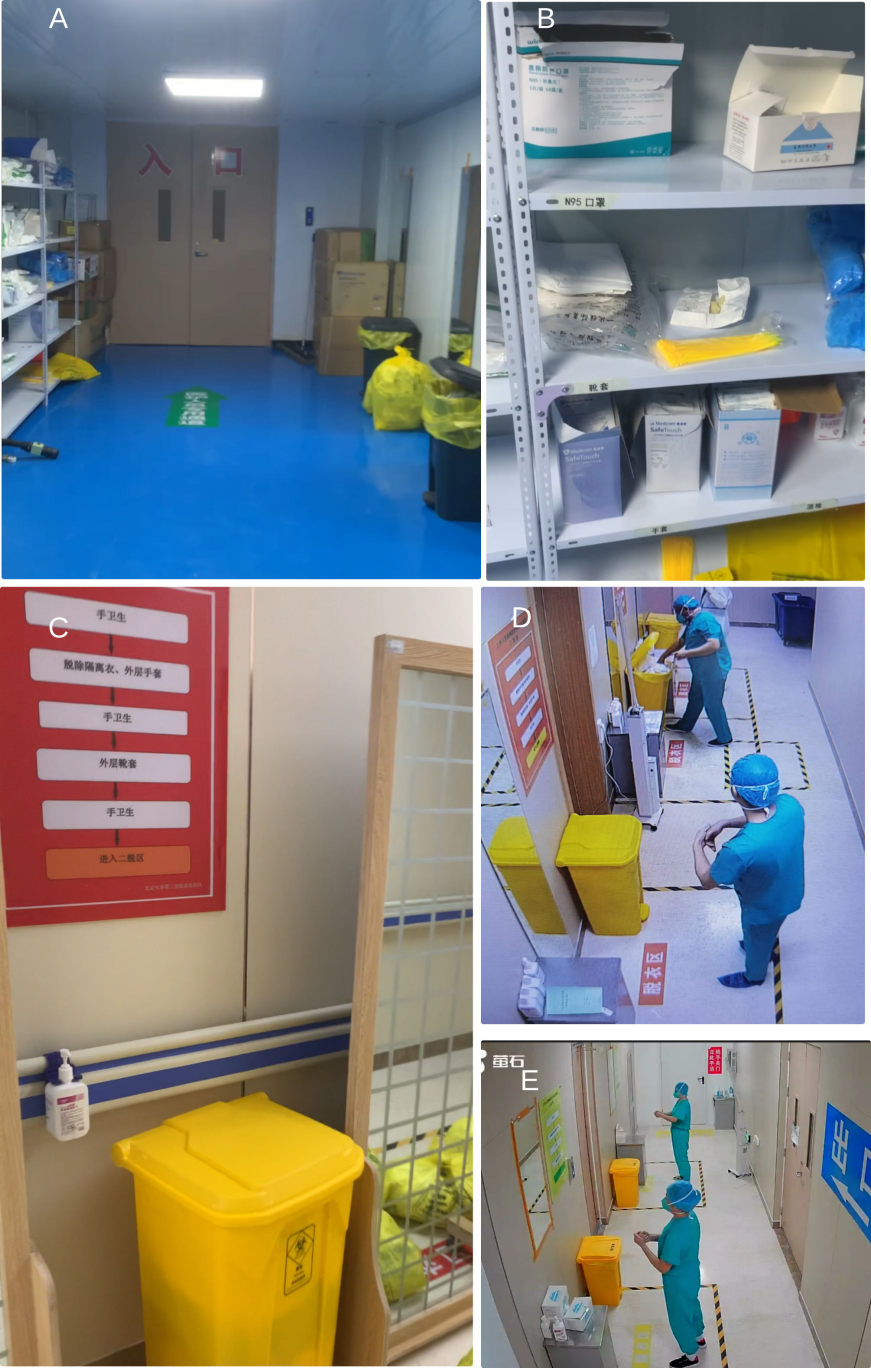
Photos of the personal protection equipment (PPE) donning and doffing areas of the hospital. The overview (A) of the donning area with (B) details of the PPE on shelves. The 3 doffing areas are shown in (C-E).

**Figure 3.**
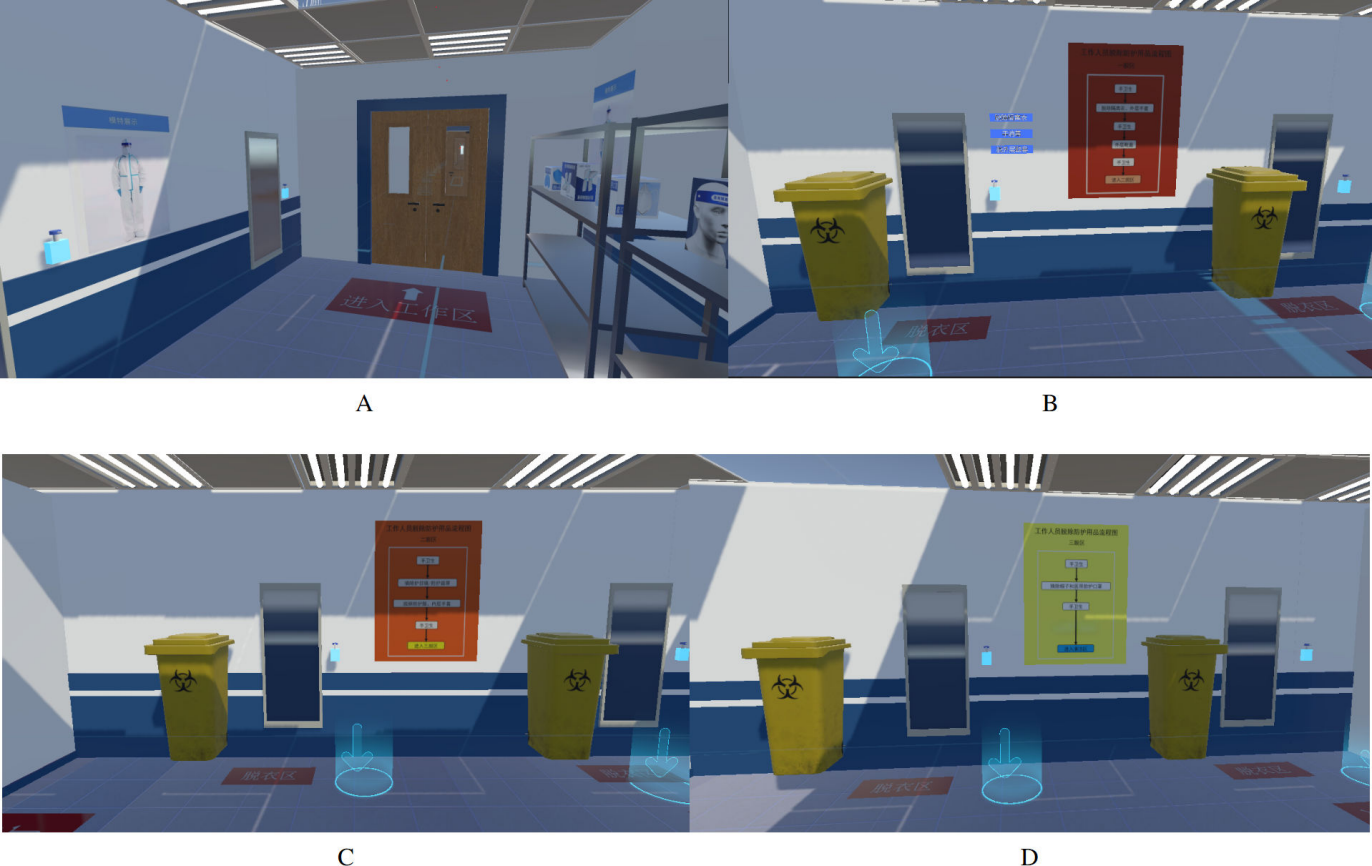
Virtual scenes of (A) the donning area and (B-D) 3 doffing areas.

Based on photos and videos of the environments of the actual hospital as shown in Figure 2, we create a virtual environment by reconstructing the rooms, items, and their arrangements (R1, R2). Four rooms are created: 1 for donning (Figure 3A) and 3 for doffing (Figure 3B-D), and the rooms are divided by doors. Other areas including the work zone of the hospital are omitted. In the donning room, PPE is placed on shelves in the order of the actual placement in the real hospital. Hand sanitizers, mirrors, and trash bins are placed at their corresponding locations. Indication signs (in Chinese), for example, floor signs “to the work zone” in the donning area (Figure 3A), “doffing zone” and instructions for doffing orders on the walls of doffing areas (Figure 3).

Teleportation zones are designated to facilitate moving to specific areas and prevent participants from accidentally exiting the room boundaries. To make the teleportation more effective, anchor points are placed in front of hand sanitizers and shelves so that users can conveniently reach target areas and items (semitransparent circles with arrows in blue off Figure 3).

#### PPE Donning and Doffing Design

Mechanisms are designed for practicing the PPE donning and doffing ordering (R1) which can be conveniently configured according to actual situations of hospitals (R3).

##### Avatar

To increase the realism of the training, an avatar is designed (R2). The camera associated with the VR headset is aligned with the eye position of the avatar for a synchronized first-person perspective. Hand controllers are bound together with the hands of the avatar, facilitating an intuitive representation of hand movements. The avatar is skeletal rigged and inverse kinematics is used to simulate human movements, for example, the movements of the whole arms when PPE is picked up, being donned, or doffed. Upon equipping and removing each PPE item, the avatar is updated visually as shown in Figure 1B.

##### Donning

**Figure 4.**
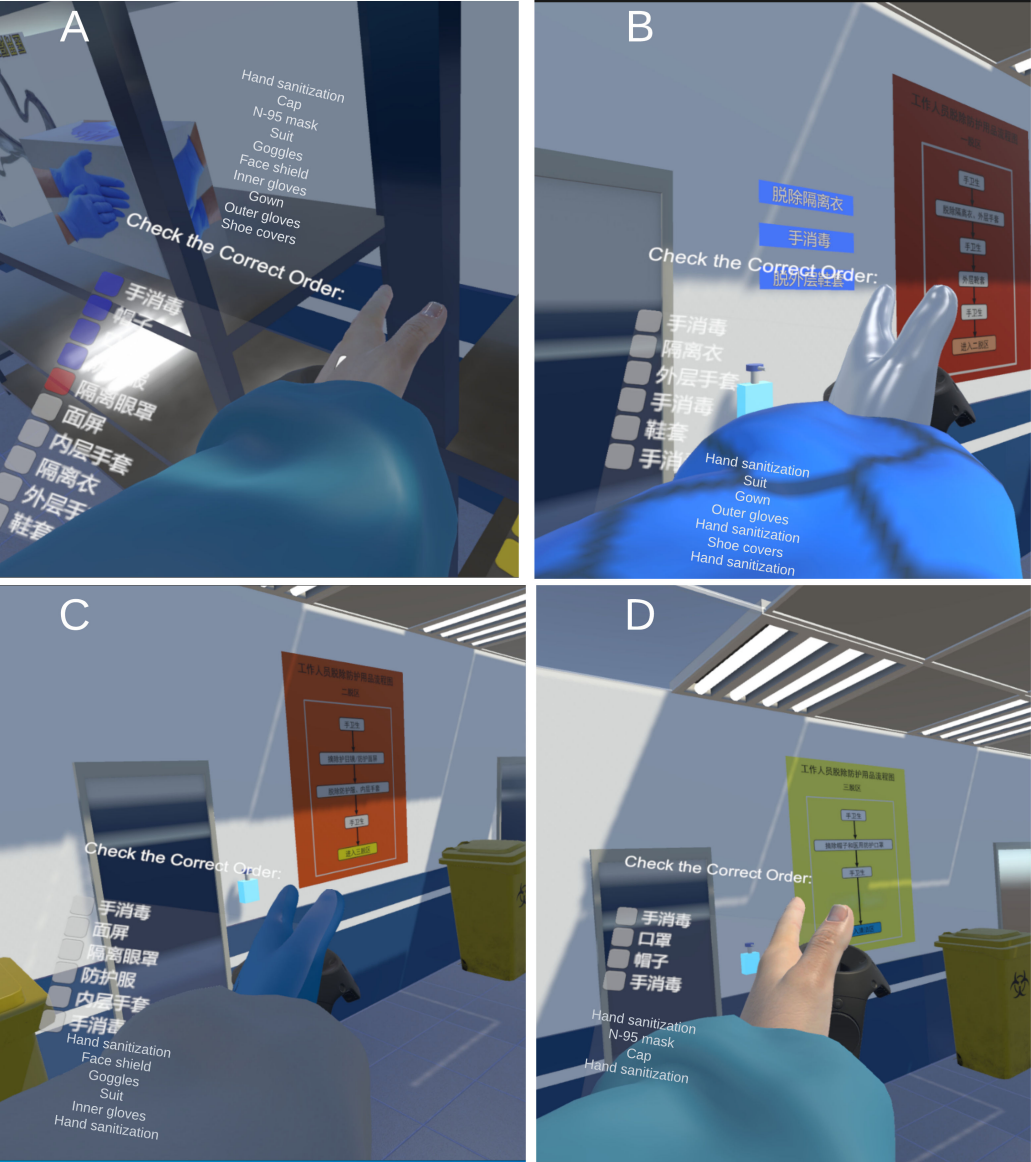
The user interface (UI) instructions of (A) the donning area and (B-D) 3 doffing areas.

The donning order is shown as an item checklist in a user interface (UI) above the left-hand controller model (Figure 4). This UI displays the sequence for donning or doffing gear within this zone using checkboxes: the box in white that the action is pending, blue signifies a completed action, and red indicates that the action was performed incorrectly as shown in Figure 4A. In the donning zone, interactable boxes of PPE are distributed on shelves. The trainee uses the controller to touch a box and presses the trigger button to grasp the PPE from the box. PPE model must be brought close to the relevant body part to simulate the donning process, for example, goggles must be near the head, while a protective suit must be near the torso. When sufficiently close, the material of the protective item turns green, indicating correct placement. Releasing the trigger button causes the item to automatically adhere to the designated position on the body.

Following the correct donning, the indicator on the left-hand UI panel is updated to blue (Figure 4A). The right-hand UI tracks each action taken during the donning and doffing process, including any erroneous movements. After the entire donning process is complete, the door of the donning zone opens, allowing the user to proceed to the next section. Note that the work zone is omitted as it is irrelevant to our training.

##### Doffing

Similar to the donning section, upon entering the doffing zones, the UI on the left controller shows the sequence for removing protective gear, along with indicative markers (Figure 4B-D). In this zone, users are required to follow the displayed instructions carefully, removing the protective items from specified parts of the body. For example, by approaching the head with the controller, the face shield can be grasped and lifted off; by approaching the torso, the protective suit can be taken off. The instruction flowchart placed on the wall within each doffing zone provides participants with guidance on the correct order for removing protective equipment as in the real hospital.

### Trajectory Recording and Visualization

#### Overview

To support the analysis of the proficiency of trainees and also potential improvements in the hospital, our VR method supports the recording of trajectories and the visualization thereafter. Trajectories of the user’s head, left hand, and right hand are recorded during the entire duration of use. Furthermore, the time spent by the user in each designated area, as well as the total time consumed, are also recorded.

**Figure 5.**
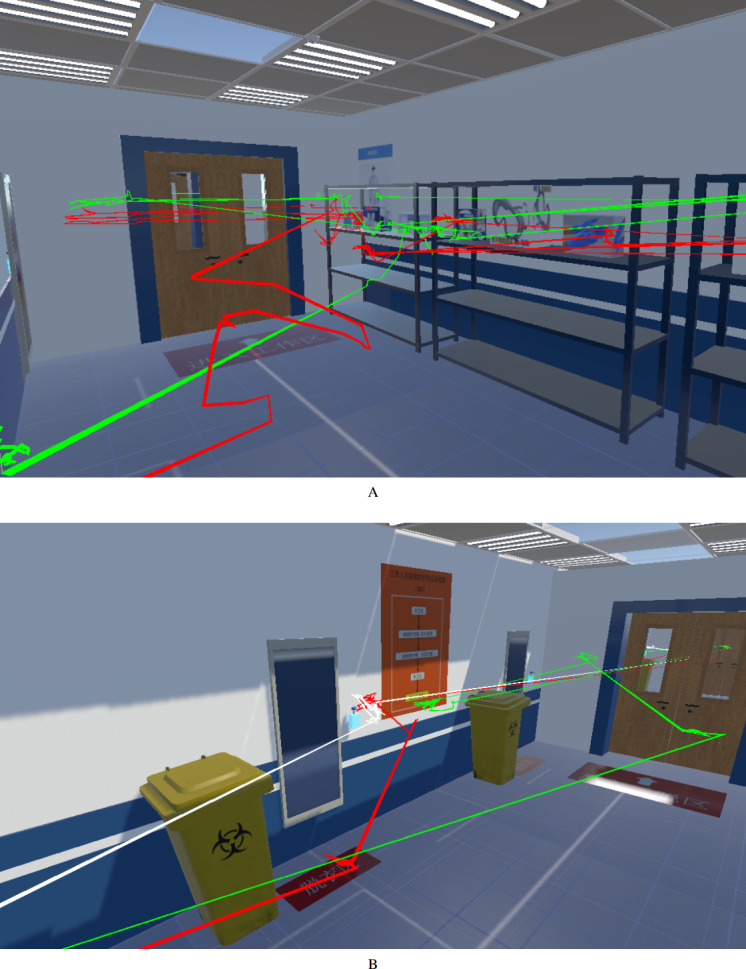
Visualization of trajectories of users in (A) the donning zone and (B) the first doffing zone after a training session.

After the training session, the recorded trajectories of head positions can be visualized for analysis as shown in Figure 5. It can be seen that trajectories vary for different users quite a lot. This is probably due to their proficiency degrees and personal preferences.

#### Pilot Study

We evaluated the effectiveness of our method in a controlled randomized trial.

### Study Design

We used a between-subject design to test 2 training conditions: our immersive PPE training (VR group), and the instructional video training (video group). To compensate for the weight and experience of a head-mounted display (HMD), both training methods are tested through HMDs [29,30].

We hypothesize that “the VR group has a comparable accuracy of memorizing the PPE donning and doffing event orders to the video group, but the usability and subjective ratings of the VR group are higher than the video group.”

### Ethical Considerations

The study was approved by the ethics review board of Peking University under the approval number IRB00001052-23206. The ethics approval covered all aspects of the study, including participant recruitment, risk management, confidentiality, data collection, and analysis. This study did not measure any direct health outcomes and thus did not require registration.

Participants were required to fill out an informed consent form prior to the study. The form outlined the procedures, potential risks, and benefits of participation, and stated that participation was voluntary and that participants could withdraw from the study at any time. Data were stored anonymously in a local repository following the data security protocol. Participants received a monetary reward of RMB ¥30 (approximately US $4) regardless of whether they completed the study successfully.

### Stimulus and Apparatus

We created an instructional video of approximately 5 minutes as no instructional video was readily available for the PPE training in the hospital of the 2022 Winter Olympic Games. We edited parts of a PPE donning and doffing training video released by the National Health Commission to the same order as the VR group; PPE doffing zoning was instructed using flowcharts that are identical to those on walls in the virtual and real environments. An HTC VIVE Pro Eye HMD and an accompanying PC with Intel i9 3.5GHz CPU, NVidia RTX 3080 GPU, and 64GB RAM were used for the study.

### Tasks

The task for participants was to memorize the event orders of donning and doffing and answer a questionnaire about details of the ordering after the training with the assigned condition. Subjective evaluations of the test condition were acquired through the National Aeronautics and Space Administration Task Load Index (NASA-TLX) [31], and the System Usability Scale (SUS) [32] questionnaires.

To assess memory of the sequence, the questionnaire contains 10 multiple-choice questions on the order of PPE and hand hygiene operations as follows.

1. Which item of protective equipment should be donned first?
2. After donning the inner gloves, what is the next piece of equipment to wear?
3. What is the next step after donning the suit?
4. In which area does the process of doffing the suit commence?
5. At which stage in the donning process are goggles worn?
6. When are boot covers typically donned in the sequence?
7. What is the last PPE to be removed within the first doffing area?
8. Which piece of equipment is typically removed first in the first doffing area?
9. Which item is removed in the final doffing area?
10. After removing the goggles and the face shield in the second doffing area, which piece of equipment should be doffed next?

Each question is worth 10 points and has 4 choices: 1 correct answer and 3 distractors, and the total score of the memorization questionnaire is 100.

### Participants

The number of participants, that is, the sample size n, of one group was determined using the 2-tailed power analysis:


$$n=\frac{2\sigma^{2}{\left(Z_{1-\alpha }+Z_{1-\beta }\right)}^{2}}{\delta^{2}}$$

Here, the test significance α is set to 0.05, and power 1-β is set to 80%. Therefore, $Z_{1-\alpha }=Z_{0.95}=1.96$, and $Z_{1-\beta }=Z_{0.8}=0.84$. We estimated that the VR group had a slightly higher mean value than the video group in tests but the variance in participants made both groups comparable. Therefore, we guessed that the scores are close, for example, 80/100 versus 70/100 making δ=(80-70)/100=0.1, and an estimated variance of σ=10/100=0.1 was used. As a result, n is 15.68 which rounds to 16. Therefore, a total of 32 participants were needed for our experiment which is in line with typical VR or mixed reality studies in medical education [19,33,34].

Participants were recruited via posters published on web forums of the institute (Peking University Health Science Center) of the first author. Participants were required to be medical students who had no previous experience with PPE training and be able to perform actions as directed by the research team. Additionally, they were required to have normal or corrected-to-normal visual acuity and adequate head and body mobility, ensuring that physical conditions did not adversely affect the experimental process. We successfully recruited 32 volunteers (16 females) aged between 18 and 30 years (mean 22.53, SD 2.65) for the study.

### Study Procedure

Before the study, each participant received a comprehensive overview of the project and provided informed consent. The participants had to sign the consent to confirm their willingness to participate in the experiment. Participants were told that they could quit the study at any time, but none of them did.

Upon agreeing to participate, each participant was randomly assigned to either the VR group or the video group. The study began with an introductory training session of approximately 10 minutes for participants to familiarize themselves with the necessary user interactions. Those in the video group were instructed on how to control video playback, pause, and adjust the progress bar using the controllers while wearing the HMD (HTC Vive Eye Pro), ensuring they completed the entire instructional video. For participants in the VR group, initial guidance via a slideshow presentation was given, followed by hands-on training within the VR setting, where they learned to navigate spatially with the controllers and perform the motions of donning and doffing PPE. A researcher offered on-site direction to assist participants through the entire sequence of the experiment—from entering the donning area to exiting the doffing zones.

Next, a free practice session of 10 minutes began, and participants were told to practice and memorize the sequence of handling PPE as well as they could. The video learning group was granted the ability to rewatch instructional videos to reinforce their retention. In contrast, the VR group was engaged in interactive “think-aloud” exercises within the immersive environment, where they independently carried out the PPE donning and doffing, but could ask any questions to the researcher. Among these questions, most were about interactions in VR, for example, how to use the controllers to check the order list, how to don shoe covers, and a few were about the event order mechanism in the simulation, but none was about the donning and doffing process itself.

Next, participants were asked to complete 1 objective questionnaire and 2 subjective questionnaires to evaluate the effectiveness of the training. Finally, a brief interview session was conducted to elicit authentic feedback and valuable suggestions from the participants regarding their experience. The average completion time of the study by a participant was 30 minutes. Each participant was compensated with 30 RMB (approximately US $4).

### Data Collection

We collected the trajectory data, questionnaires, and useful comments from the participants.

The trajectory data are potentially useful for analyzing the behavior of participants, and improving the arrangement of items and the allocation of areas for a more efficient workflow. The positional data of the HMD and both hand controllers of each user were recorded to construct trajectory profiles, at a sampling rate of 2 Hz to prevent latency during the VR training session. In addition to the questionnaire, we collected basic demographics of participants and their prior experience with VR.

## Results

### Overview

**Figure 6.**
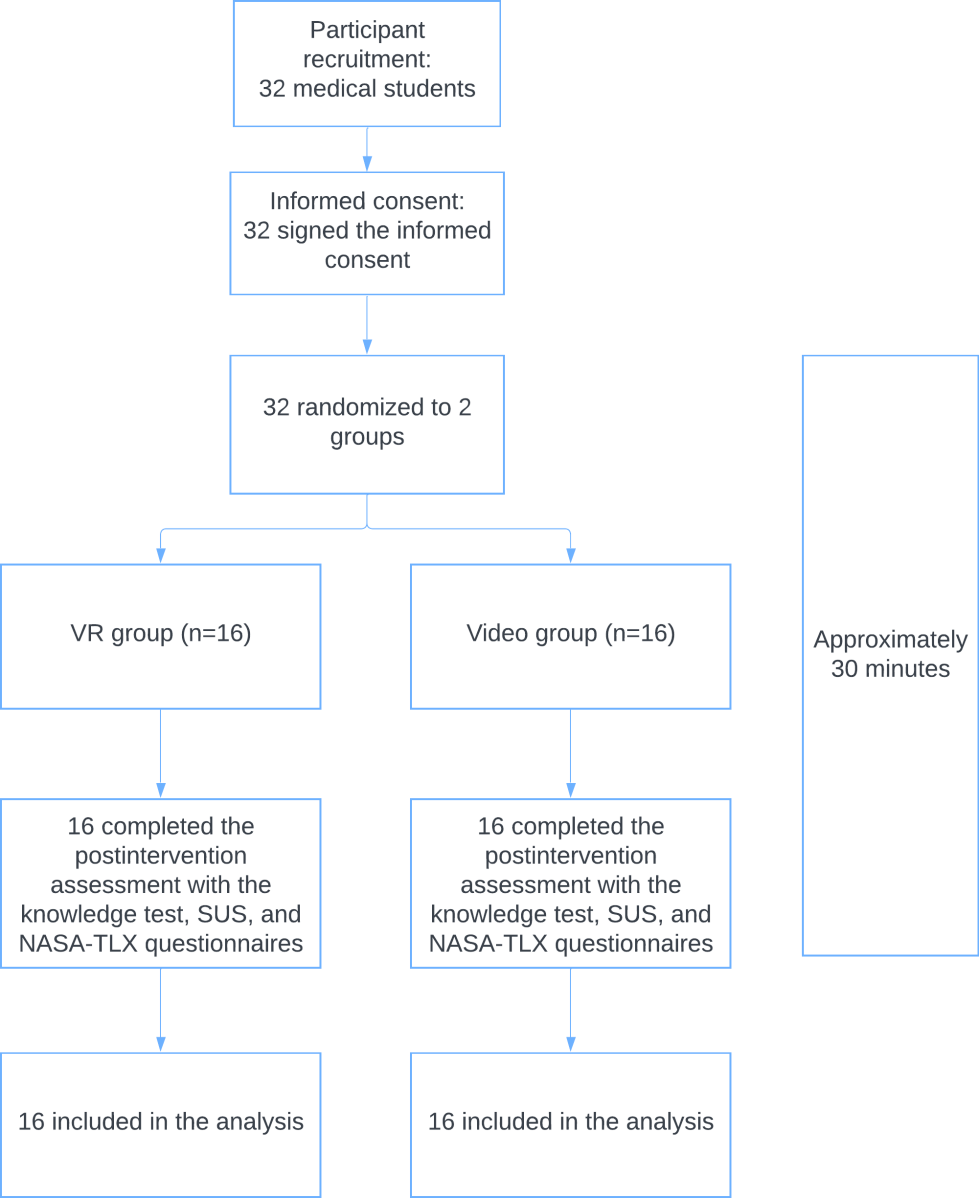
Trial flow chart. NASA-TLX: National Aeronautics and Space Administration; SUS: System Usability Scale.

**Figure 7.**
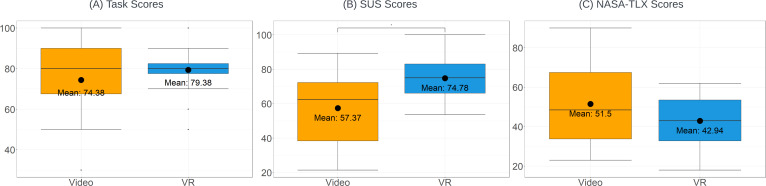
Boxplots of (A) the task scores, (B) SUS scores, and (C) NASA-TLX. NASA-TLX: National Aeronautics and Space Administration Task Load Index; SUS: System Usability Scale.

The flow diagram of the trial is shown in Figure 6. The performance of the PPE event order memorization task, usability, task workload, and virtual trajectories are analyzed for the study. Results of the study are summarized in Table 1 and visualized as boxplots as shown in Figure 7.

**Table 1.** Results of questionnaires of the pilot study. *P* values of Student *t* test between the 2 groups are also reported.

	VR^a^ (N=16), mean (SD)	Video (N=16), mean (SD)	*P* value (*t* test)
Task scores	79.38 (12.89)	74.38 (17.88)	.37
SUS^b^	74.78 (13.58)	57.37 (21.13)	.009
NASA-TLX^c^	42.94 (13.01)	51.50 (20.44)	.17

a VR: virtual reality.

b SUS: System Usability Scale.

c NASA-TLX: National Aeronautics and Space Administration Task Load Index.

### Event Order Memorization

Descriptive statistics of the event order memorization questionnaire are shown in the boxplot in Figure 7A. The VR group has a higher mean, equal median, and lower variance compared with the video group.

A Shapiro-Wilk normality test shows that both groups follow the assumption of normality (*P*=.17 for the VR group, *P*=.08 for the video group). Levene test (*F*_1, 30_ =2.258, *P*=.14) shows that the equal variance assumption is met. The intergroup differences are then tested with a Student *t* test that shows no significance (*P*=.37).

### System Usability Scale

The SUS scores of the VR group have a mean of 74.78 (SD 13.58) and a rather small variance. The boxplot of SUS scores can be seen in Figure 7B. The VR group has a higher mean and median, and a smaller variance, compared with the video group.

Both groups satisfy the normal distribution criteria with the Shapiro-Wilk test (W(16)=0.961, *P*=.67 for the VR group, and W(16)=0.926, *P*=.21 for the video group). Levene test demonstrates no significant disparity between the 2 groups (*F*_1, 30_=2.990, *P*=.09), therefore, it is reasonable to deduce that variances are equivalent across the groups. A subsequent *t* test shows a statistical significance (*P*=.009) between the 2 groups.

### NASA-TLX

The NASA-TLX result is shown in Figure 7C: the VR group has a lower mean and median, and a smaller variance compared with the video group. The NASA-TLX scores of both groups follow the normal distribution criteria after the Shapiro-Wilk test (W(16)=0.956, *P*=.60 and W(16)=0.962, *P*=.69, for VR and video groups, respectively), and show no significant differences in variance with the Levene test (*F*_1, 30_=3.752, *P*=.06). No significant difference between the groups is found with the *t* test (*P*=.17).

### Trajectory Analysis

**Figure 8.**
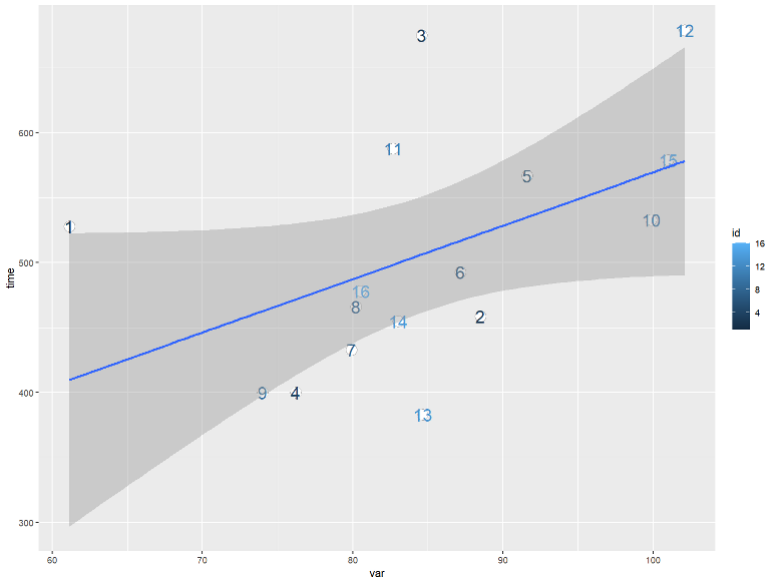
The scatterplot of the total variance (var) of trajectories and task completion time (time) with the linearly fitted trend.

For quantitative analysis, we calculated the sum of the gradient magnitude of a trajectory as the total variance to describe the degree of fluctuation. A scatterplot of total variance versus task completion time is shown in Figure 8, where the user ID is drawn on data points. A positive correlation (Pearson correlation coefficient=0.47) is observed between the variance and time as shown in the blue fitted line in Figure 8.

**Figure 9.**
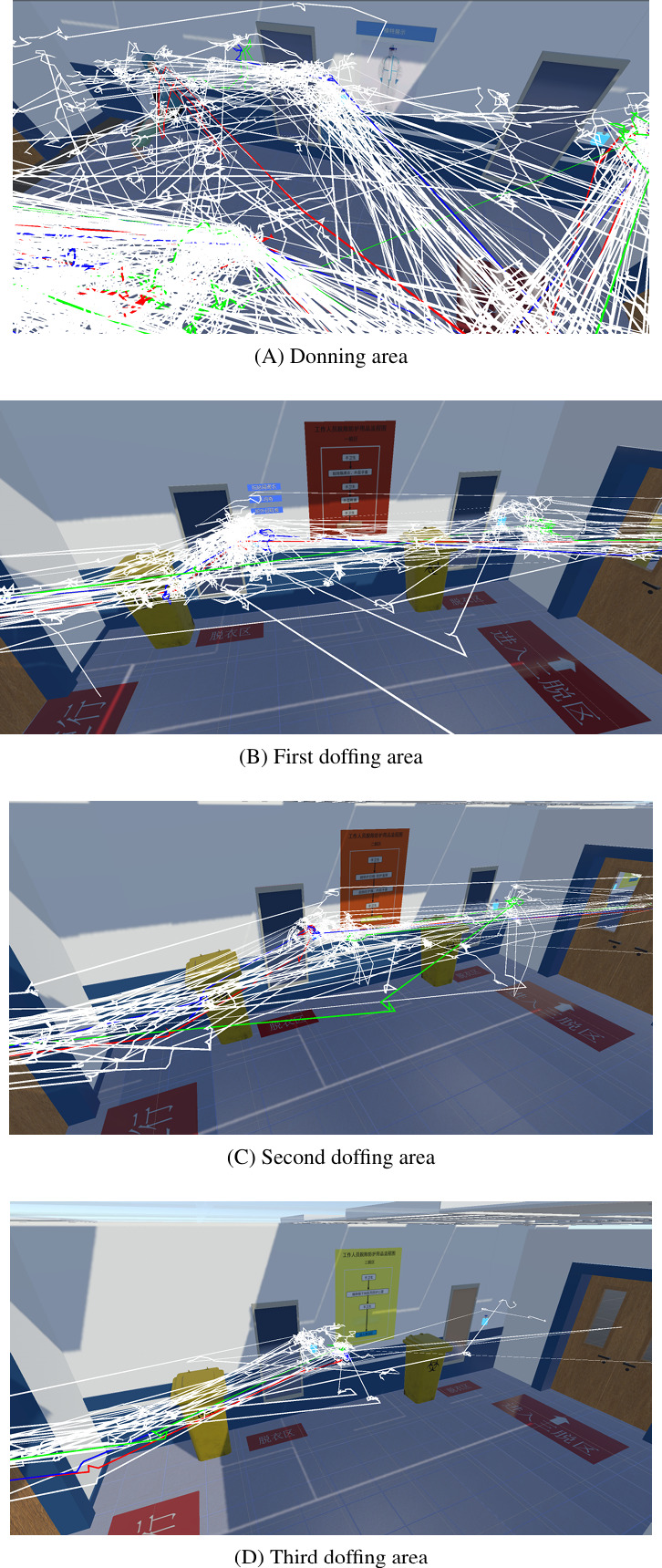
Visualization of trajectories of the participants as spaghetti plots.

Moreover, we visualize the actual trajectory of each user within the Unity scene, as shown in Figure 9. It can be seen that the trajectories are more cluttered in the donning area (Figure 9A) than in the doffing areas (Figure 9B-D). We identified some trajectories that are long and with few fluctuates (colored), and these correspond to participants who were fluent in VR.

## Discussion

### Main Findings

The VR group has a higher mean task score and a smaller variance than the video group. However, the *t* test on order task scores of the 2 groups is not significant.

One possible explanation for the similar learning outcomes is that participants in both groups used strategies to enhance their learning intentionally, as they were told to memorize the event order. Participants in the video group repeatedly watched clips of unfamiliar sequences, while those in the VR group read and memorized the order presented on the UI and the instructions displayed on the walls. Another factor is that the advantages of the immersiveness of the VR group and the familiarity of the real spatial settings thereof may not have been fully captured through the questionnaire.

For usability, it is important that the mean SUS score of the VR group (74.78, SD 13.58) exceeds the threshold of generally accepted systems [35] indicating above-average usability. This is because the mechanism and interactions of the simulation were iteratively designed and tested for usability.

The VR group also has significantly better usability than the video group. Our explanation is that in VR, the relatively small buttons of the video player had to be clicked remotely through rays cast from controllers require high accuracy. Compared with the ease of using a mouse or keyboard outside of VR, the usability is low. In addition, participants in the VR group were limited to simple interactions, which made them feel less engaged and resulted in lower subjective usability ratings. However, we also note that the simplicity of interactions in the video group makes its usability assessment of only secondary importance.

With the NASA-TLX scores, it can be inferred that the psychological workload or task load required by participants for both training methods was roughly equal for the case of memorizing donning and doffing event orders. The smaller variance suggests that the VR experience was more consistent across participants compared with the video group. The higher mean and larger variance observed in the video group can be attributed to their less engaging scenario and fewer interactions. Participants in this group focused only on memorizing the order information, perceiving the study mainly as a test. This focus may have induced more pressure compared with the VR group, which could lead to an increased perceived workload and, consequently, a higher mean. Additionally, the reactions of participants under pressure were more varied when compared with those in the VR group.

For the analysis of trajectories of the VR group, the positive correlation between total variance and time suggests that participants who tended to move around spent more time to complete. The visualization of trajectories reveals that participants took a relatively long time to get familiar with the environment, searched for PPE items and donned them, and moved back and forth to use the hand sanitizer. Unlike in the real environment, the simulation faced a number of design and implementation challenges: the sensitivity of collision detection required participants sometimes to make several attempts in the simulation to interact with PPE items; also, the mechanism does not support the participants to collect all PPE items at once and don them in one place in front of the mirror. We believe that all these factors contribute to the cluttering in the donning area.

Our trajectory visualization does not only provide insights into the simulation but also, more importantly, identifies potential issues in the real environment and helps nosocomial infection control experts make improvements. For instance, large labels of the donning order of each PPE, for example, 1 for the cap in our case, can be clearly placed on the shelf and the wall behind it in the donning area to reduce the searching time. Pictograms of the donning and doffing orders can be placed on walls in complement to text instructions to reduce the cognitive load.

Given the study results and the analysis, we conclude that the VR group has an above-average usability which is significantly better than the video group, and both groups have comparable donning and doffing event order memorization task scores, and task workload. Therefore, our hypothesis can be accepted.

### Feedback

During the training, all participants noted that their initial focus was on familiarizing themselves with the interactions. It was only during the free practice phase that they shifted their attention to memorizing the sequence of donning and doffing. This suggests a need to reinforce operational guidance in the early training phase and to emphasize memory training exercises in the subsequent practice sessions. Most VR participants (14/16) indicated that they relied on the left-hand UI guidance bar to remember the sequence of steps. However, 2 participants suggested that providing a flow diagram in the donning area, similar to that in the doffing areas, could potentially improve the efficiency of memorization.

The majority of participants in the VR group reported a superior experience to those of the video group, highlighting increased engagement, satisfaction, and interest provided by this gamification method. They agreed that the spatial context in the virtual environment can improve their confidence in task performance and reduce the adaptation time in a real hospital. They also commented that the customizable donning and doffing order makes our VR method a generic and flexible tool that can adapt to a wide range of PPE training scenarios of varying prevention levels.

### Limitations

Several limitations are identified for this study. The use of hand-held controllers instead of hand-tracking added to the overhead of the VR method and reduced its efficiency. The allocation to different groups may potentially introduce a bias in the satisfaction, and performance of participants.

The mean values of test scores and task load of the VR group were both better than those of the video group, but they were not of statistical significance suggesting that the selected sample size may be increased. A larger sample size would be helpful to show the existence or absence of significance of differences in task scores and task loads.

Our VR method does not currently support the creation of a customizable immersive space with room modules, for example, having fewer or more doffing areas. Simulating key steps during donning or doffing PPE with high-precision tracking of movements is not possible with our method. Finally, our method does not yet simulate emergent contamination situations for more comprehensive PPE training.

### Conclusions and Future Work

We have presented a PPE training method in VR with a flexible donning and doffing ordering and spatial context. In immersive virtual environments that resemble the relevant configurations of an actual hospital, trainees can practice customized PPE donning and doffing in the corresponding zones to aid the memorization of the sequence and familiarize them with the hospital settings. With a pilot study (N=32), the method is compared against the video-based training in a randomized controlled trial. Results suggest that our new method yields a comparable memorization performance and cognitive load to the video training, but higher SUS scores, satisfaction, and engagement. Our method provides an immersive experience that is not possible with video training. It is important to note that our work does not intend to replace but to complement the existing training. Hands-on PPE donning and doffing in the real world is irreplaceable for successful infection prevention and control.

In the future, we will extend the functionalities of our VR method in several ways and conduct comparative experiments to study its effectiveness. We will extend our tool for the training of hazardous operations under personal protection for different roles in a hospital. We would like to cover more hospital areas and reconstruct realistic scenes captured using neural radiance field techniques [36] for improved realism. We also plan to support the training for detailed donning and doffing actions using real motions instead of controllers with the aid of an optical motion tracking system. For comparative studies in the future, we would like to include larger and more diverse participant groups.

## Supplementary material

10.2196/69021 Checklist 1 CONSORT-eHEALTH checklist (V 1.6.1).

## Abbreviations

HMD: head-mounted display
NASA-TLX: National Aeronautics and Space Administration Task Load Index
PPE: personal protection equipment
R1: requirement 1
R2: requirement 2
R3: requirement 3
SUS: System Usability Scale
UI: user interface
VR: virtual reality

## References

[1] Kirkland KB, Briggs JP, Trivette SL, Wilkinson WE, Sexton DJ (1999). The impact of surgical-site infections in the 1990s: attributable mortality, excess length of hospitalization, and extra costs. Infect Control Hosp Epidemiol.

[2] Haque M, Sartelli M, McKimm J, Abu Bakar M (2018). Health care-associated infections - an overview. Infect Drug Resist.

[3] Pittet D, Allegranzi B, Sax H (2006). Evidence-based model for hand transmission during patient care and the role of improved practices. Lancet Infect Dis.

[4] Stone PW, Pogorzelska-Maziarz M, Herzig CTA (2014). State of infection prevention in US hospitals enrolled in the National Health and Safety Network. Am J Infect Control.

[5] John A, Tomas ME, Cadnum JL (2016). Are health care personnel trained in correct use of personal protective equipment?. Am J Infect Control.

[6] Haegdorens F, Franck E, Smith P, Bruyneel A, Monsieurs KG, Van Bogaert P (2022). Sufficient personal protective equipment training can reduce COVID-19 related symptoms in healthcare workers: a prospective cohort study. Int J Nurs Stud.

[7] Saraswathy T, Nalliah S, Rosliza AM (2021). Applying interprofessional simulation to improve knowledge, attitude and practice in hospital- acquired infection control among health professionals. BMC Med Educ.

[8] (2020). Notice on improving the prevention and control of infection in fever outpatient and medical institutions [Report in Chinese]. http://www.nhc.gov.cn/yzygj/s3573d/202006/4e456696ceef482996a5bd2c3fb4c3db.shtml.

[9] Chang Y, Ai Z, Wargocki P, Liu Y, Hu Y (2024). Design of convertible patient care unit for both non-pandemic and pandemic times: prototype, building spatial layout, and ventilation design. Build Environ.

[10] Mumma JM, Durso FT, Ferguson AN (2018). Human factors risk analyses of a doffing protocol for ebola-level personal protective equipment: mapping errors to contamination. Clin Infect Dis.

[11] Díaz-Guio DA, Ricardo-Zapata A, Ospina-Velez J, Gómez-Candamil G, Mora-Martinez S, Rodriguez-Morales AJ (2020). Cognitive load and performance of health care professionals in donning and doffing PPE before and after a simulation-based educational intervention and its implications during the COVID-19 pandemic for biosafety. Infez Med.

[12] Yu K, Roth D, Strak R Mixed reality 3D teleconsultation for emergency decompressive craniotomy: an evaluation with medical residents.

[13] Chheang V, Schott D, Saalfeld P (2024). Advanced liver surgery training in collaborative VR environments. Comput Graph.

[14] Heinrich F, Joeres F, Lawonn K, Hansen C (2019). Comparison of projective augmented reality concepts to support medical needle insertion. IEEE Trans Visual Comput Graphics.

[15] Heinrich F, Schwenderling L, Joeres F, Lawonn K, Hansen C (2020). Comparison of augmented reality display techniques to support medical needle insertion. IEEE Trans Visual Comput Graphics.

[16] Allgaier M, Huettl F, Hanke L LiVRSono - virtual reality training with haptics for intraoperative ultrasound.

[17] Zhao G, Fan M, Yuan Y, Zhao F, Huang H (2021). The comparison of teaching efficiency between virtual reality and traditional education in medical education: a systematic review and meta-analysis. Ann Transl Med.

[18] Kavanagh S, Luxton-Reilly A, Wuensche B, Plimmer B (2017). A systematic review of virtual reality in education. Theme Sci Technol Edu.

[19] Birrenbach T, Zbinden J, Papagiannakis G (2021). Effectiveness and utility of virtual reality simulation as an educational tool for safe performance of COVID-19 diagnostics: prospective, randomized pilot trial. JMIR Serious Games.

[20] Buyego P, Katwesigye E, Kebirungi G (2022). Feasibility of virtual reality based training for optimising COVID-19 case handling in Uganda. BMC Med Educ.

[21] Yu M, Yang M, Ku B, Mann JS (2021). Effects of virtual reality simulation program regarding high-risk neonatal infection control on nursing students. Asian Nurs Res (Korean Soc Nurs Sci).

[22] Yu M, Yang MR (2022). Effectiveness and utility of virtual reality infection control simulation for children with COVID-19: quasi-experimental study. JMIR Serious Games.

[23] Masson C, Birgand G, Castro-Sánchez E (2020). Is virtual reality effective to teach prevention of surgical site infections in the operating room? Study protocol for a randomised controlled multicentre trial entitled VIP room study. BMJ Open.

[24] Omori K, Shigemoto N, Kitagawa H (2023). Virtual reality as a learning tool for improving infection control procedures. Am J Infect Control.

[25] Kakdas YC, Demirel D, Barker JR Virtual reality-based donning and doffing simulator.

[26] Kakdas YC, Kockara S, Halic T, Demirel D (2024). Enhancing medical training through learning from mistakes by interacting with an ill-trained reinforcement learning agent. IEEE Trans Learn Technol.

[27] Kravitz MB, Dadario NB, Arif A (2022). The comparative effectiveness of virtual reality versus e-module on the training of donning and doffing personal protective equipment: a randomized, simulation-based educational study. Cureus.

[28] Tsukada K, Yasui Y, Miyata S (2024). Effectiveness of virtual reality training in teaching personal protective equipment skills. JAMA Netw Open.

[29] Skreinig LR, Kalkofen D, Stanescu A (2023). guitARhero: interactive augmented reality guitar tutorials. IEEE Trans Visual Comput Graphics.

[30] Haltner P, Goddy-Worlu R, Forren J, Nicholas C, Reilly D A comparative evaluation of AR embodiments vs. videos and figures for learning bead weaving.

[31] Brooke J (1996). Usability Evaluation In Industry.

[32] Hart SG, Staveland LE, Hancock PA, Meshkati N (1988). Human Mental Workload.

[33] Bogomolova K, van der Ham IJM, Dankbaar MEW (2020). The effect of stereoscopic augmented reality visualization on learning anatomy and the modifying effect of visual‐spatial abilities: a double‐center randomized controlled trial. Anatomical Sciences Ed.

[34] Ho S, Liu P, Palombo DJ, Handy TC, Krebs C (2022). The role of spatial ability in mixed reality learning with the HoloLens. Anat Sci Educ.

[35] Bangor A, Kortum PT, Miller JT (2008). An empirical evaluation of the system usability scale. Int J Hum Comput Interact.

[36] Mildenhall B, Srinivasan PP, Tancik M, Barron JT, Ramamoorthi R, Ng R NeRF: representing scenes as neural radiance fields for view synthesis.

